# Nanotechnology in Kidney and Islet Transplantation: An Ongoing, Promising Field

**DOI:** 10.3389/fimmu.2022.846032

**Published:** 2022-04-08

**Authors:** Wei Wang, Ya Teng, Ji-Ji Xue, Hong-Kai Cai, Yu-Biao Pan, Xing-Nan Ye, Xin-Li Mao, Shao-Wei Li

**Affiliations:** ^1^ Taizhou Hospital of Zhejiang Province Affiliated to Wenzhou Medical University, Linhai, China; ^2^ Taizhou Hospital of Zhejiang Province, Zhejiang University, Linhai, China; ^3^ Taizhou Hospital of Zhejiang Province, Shaoxing University, Linhai, China; ^4^ Key Laboratory of Minimally Invasive Techniques and Rapid Rehabilitation of Digestive System Tumor of Zhejiang Province, Taizhou Hospital of Zhejiang Province Affiliated to Wenzhou Medical University, Linhai, China; ^5^ Department of Gastroenterology, Taizhou Hospital of Zhejiang Province Affiliated to Wenzhou Medical University, Linhai, China; ^6^ Institute of Digestive Disease, Taizhou Hospital of Zhejiang Province Affiliated to Wenzhou Medical University, Linhai, China

**Keywords:** nanotechnology, kidney, islet, transplantation, promising field

## Abstract

Organ transplantation has evolved rapidly in recent years as a reliable option for patients with end-stage organ failure. However, organ shortage, surgical risks, acute and chronic rejection reactions and long-term immunosuppressive drug applications and their inevitable side effects remain extremely challenging problems. The application of nanotechnology in medicine has proven highly successful and has unique advantages for diagnosing and treating diseases compared to conventional methods. The combination of nanotechnology and transplantation brings a new direction of thinking to transplantation medicine. In this article, we provide an overview of the application and progress of nanotechnology in kidney and islet transplantation, including nanotechnology for renal pre-transplantation preservation, artificial biological islets, organ imaging and drug delivery.

## Introduction

As a reliable survival choice for patients with end-stage organ failure, organ transplantation has progressed markedly in the era of modern medicine. With progress in surgical approaches, anesthesia, nursing and the addition of immunosuppressants, the survival of grafts has markedly improved. However, with the extension of human life expectancy and the increasing incidence of chronic diseases, the demand rate for organ transplantation may also increase, which will further aggravate the serious imbalance between organ demand and supply, the most important issue in transplantation medicine (1–3).

In addition to organ shortages, other challenges, such as the risk of transplantation, immune rejection, long-term immunosuppressants and their inevitable side effects, will limit the application of transplantation technology (2, 4–6). Fortunately, the combination of emerging technologies with transplantation approaches is influencing traditional transplantation methods (7), such as single-cell sequencing technology being used to determine cell heterogeneity and new molecular characteristics for immune rejection and CRISPR/cas9 gene-editing technology being used to generate transgenic pigs to resolve issues of organ shortage (8, 9).

Nanotechnology has developed rapidly in the past few decades and has its applications in many fields, including medicine. Nanomaterials are usually divided into metallic, organic and semiconducting particles, with sizes ranging from 1 to 100 nm. The main purpose of nano medicine is to diagnose and treat diseases at the nano level using the properties and properties of nano materials (10). For example, nanomaterials are used as carriers to achieve drug targeting and controlled release (5, 10), improve pharmacokinetics (11) and reduce drug toxicity and side effects (12, 13). They have also been used for direct tumor treatment (14) and molecular imaging (15). Nanomedicine has unlimited potential. Its application in transplantation medicine overcomes the obstacles of many traditional transplantation methods, such as optimizing the pharmacokinetics of immunosuppressive drugs, improving organ or tissue preservation, aiding in the generation of artificial biological organs and improving organ imaging (5, 16–18).

We focused on the outcomes of combining nanotechnology with kidney transplantation and islet transplantation and summarized the current popular applications and latest progress in this field.

## Nanotechnology in Kidney Preservation

### Implantable Artificial Kidney (IAK)

Kidney transplantation is the most promising way to cure renal failure, and dialysis is one way to extend the survival of patients who must wait because of the scarcity of kidney sources. However, dialysis inevitably transforms the daily lives of patients.

**Figure 1:**
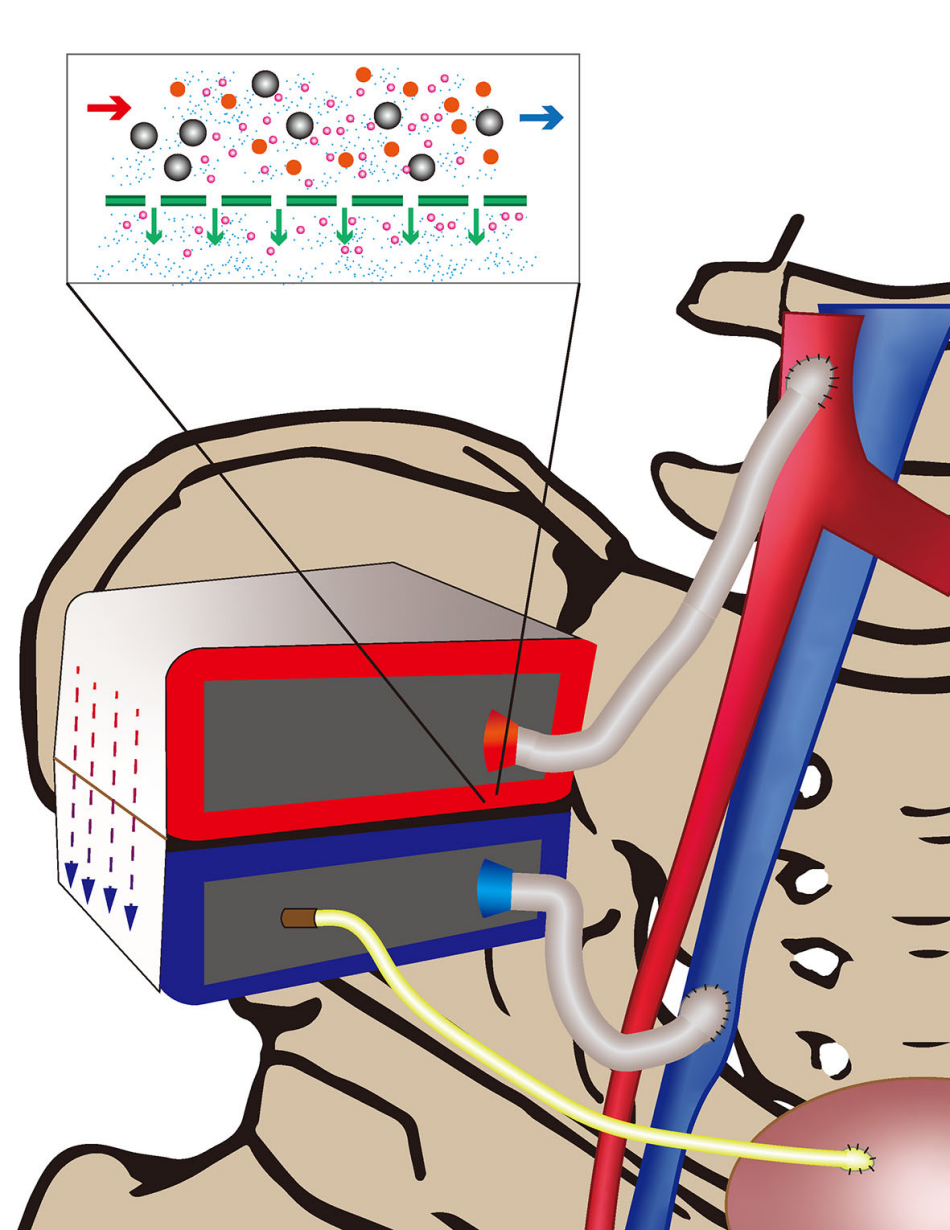
Arterial blood (red) is filtered through the Iak and the generated urine passes through a tubing (yellow) into the bladder. Filtered blood flows back into the vein (blue).

The combination of silicon nanotechnology and tissue engineering has given birth to a device that has changed the current situation: the IAK. The HemoCartridge of the IAK is responsible for filtration, and the IAK also possesses a bioreactor to culture renal tubular cell epithelium (BioCartridge). Benefiting from silicon nanotechnology, each microchip in the HemoCartridge forms a filtration channel resembling a glomerular slit diaphragm that can block molecules such as albumin and allow waste filtration of small molecules. The device, which is implanted surgically, uses blood pressure for filtering power, not relying on an electric pump and dialysate, and simulates the natural kidney function (**Figure 1**). This technology is of great significance for patients with chronic kidney disease. The implantation of an artificial kidney can greatly reduce the urgent need for renal transplantation and alleviate the current pressure of renal scarcity (19–24).

### Donated After Circulatory Death (DCD) Kidney and Its Role in Alleviating Kidney Donor Deficiency

An additional way to alleviate the pressure associated with the kidney transplant donor shortage is the acceptance of less-ideal organs, such as the gradually increasing number of kidneys that are donated after circulatory death (DCD). The issue with DCD kidneys is their longer warm ischemic time, leading to injury. The effects of such injury are diverse. The kidney is particularly prone to acute tubular necrosis, resulting in a delay in the renal function recovery, while the pancreas is more prone to graft thrombosis and islet loss (25–27). However, these should not be reasons for abandoning DCD organs. The experience with the UK DCD kidney transplantation program supports the utilization of DCD organs to help alleviate the global shortage of kidneys from deceased donors (17, 28).

### Suitability of Hypothermic Machine Perfusion (HMP) Over Static Cold Storage (SCS) for the Preservation of DCD Kidneys

Organ preservation is an important step before transplantation. At present, the main goal is to preserve the function of organs after they are removed from the body and reduce the injury of ischemia and reperfusion (29). SCS, which is easy to operate and inexpensive, has become the main method of organ preservation in solid organ transplantation. However, in the era of the increasing use of DCD organs, the preservation effect has been unsatisfactory.

Organ damage due to hypoxia as well as reperfusion is particularly pronounced in DCD organs. This has necessitated revisiting organ preservation methods that predate SCS, with a renewed emphasis on organ preservation to reduce organ damage from ischemia-reperfusion injury(IRI). A prospective study on HMP carried out by Moers et al. pulled the prelude to the use of mechanical perfusion for clinical organ preservation. Their team randomly divided the kidneys of 336 deceased donors into HMP and SCS groups and then transplanted the kidneys into 672 subjects. After one year of follow-up, it was found that perfusion reduced the incidence and extended the duration of a delayed graft function. The one-year graft survival was also better than that in the SCS group (30).

### Normothermic Machine Perfusion (NMP) as an Alternative to Machine Perfusion

Since HMP does not completely mimic the organ circulatory environment in a physiological state, the effect of cold ischemia on graft injury as well as adverse graft outcomes has been noted. Therefore, NMP, which lacks the disadvantages of cold ischemia, has attracted widespread interest (31).

Arnaud et al. performed orthotopic transplantation after NMP of isolated rabbit kidney, suggesting that this method for organ preservation may be ideal for improving the current state of DCD organ preservation (32). Not only do normothermic perfused kidneys suffer less endothelial cell damage, but the partial damage induced by cold ischemia can be reversed, and the metabolic function of the organ can be restored as well, thereby improving the final transplantation outcome (33–35). Given the good results obtained in animal trials, NMP has been used in clinical trials with marginal kidneys, further demonstrating the superiority of this approach (36, 37).

The exploration of the utility of NMP continues. After Kaths et al. found that prolonged NMP in isolated porcine kidneys resulted in better graft outcomes than either SCS alone or that combined with short-term NMP (38). Annemarie­Weissenbacher’s group were further explored in human kidney with developing an automated normothermic perfusion system. This research group found a trend toward improvement in kidney quality after prolonged perfusion(24 hours) (39).

### Graft Treatment During Machine Perfusion

Hypoxia impairs the endothelial cell function, promotes inflammation and procoagulant activity and leads to vascular convergence, which is considered key to IRI (40–42). Although these processes can be avoided to some degree with the use of unfractionated heparin, the risk of systemic bleeding must still be considered. Semisynthetic antiplatelet and anticoagulants (APACs) have been found to protect the kidney from moderate and severe IRI by reducing adaptive immune activation and vascular injury, but the systemic risk of systemic administration still needs attention (43, 44). The MP, particularly NMP is more than a preservation benefit to the graft, and more importantly, treatment and handling of the graft before transplantation can be performed during the perfusion period, further improving graft efficacy (45). It may be a more effective way to use mechanical perfusion as a vehicle combined with drugs targeting endothelial cells(ECs) to help reduce the local IR response. Hamaoui et al. used thrombalexin (TLN) for local anticoagulation during HMP, which confirmed the feasibility of HMP as a vehicle. This method promoted the adhesion between TLN and its target to improve organ perfusion (46).

### Nanotechnology Combined With Machine Perfusion, 1 + 1 > 2

A rather promising direction for combination nanotherapeutics during machine perfusion. Machine perfusion provides delivery motivation for nanotherapeutics, whereas nanotherapeutics can accomplish treatment of more targeted pretransplant grafts during machine perfusion.

Devalliere et al. used biodegradable PLGA polymer nanoparticles to wrap microRNA-132, which can promote angiogenesis. At the same time, this complex was combined with cyclic RGD (cRGD) peptides to promote its uptake by ECs, and was then implanted into ECs before transplantation, and then the collagen-fibronectin-based scaffold was used to control the transplantation site of ECs. The slowly released RNA then continuously promotes angiogenesis at the target site (47).

Nanoparticles will combine with proteins in organisms to form a coating called protein corona on the surface. This reaction will greatly hinder the normal function of nanoparticles, and may also cause the death of endothelial cells (48). The use of NPs to treat grafts during mechanical perfusion can help prevent the aggregation of NPs in the macrophage system and the “protein corona” caused by serum proteins. Even so, there is still room for improving the targeting ability of NPs. Ensuring that NPs accumulate in target cells to influence perfusion remains challenging. Tietjen et al. bound the anti-human CD31 antibody to NPs and applied it to human kidneys during the machine perfusion phase. The experimental results showed that, compared with non-targeted NPs, targeted NPs had a significantly enhanced enrichment effect, but this effect was weakened by nonspecific aggregation. The nonspecific aggregation of NPs is particularly significant in areas with insufficient blood perfusion, which is a key factor hindering the role of therapeutic NPs. But, however, it can provide direction for predicting the therapeutic effect of NPs or evaluating organ function in the future (49).

Improving the targeting effect of NPs during NMP requires not only modifying NPs but also improving the microenvironmental status of the organ itself. Areas of hypoperfusion were found to have significant microvascular obstruction. Indeed, DiRito et al. found that the microvascular obstruction in the kidney was due to fibrin (ogen) from the proximal tubular epithelium, and treatment with a tPA+plasminogen regimen prior to transplantation resulted in improved microvascular obstruction and vascular targeting capability of the NPs (50). During NMP, a large number of nanoparticles are released, including kidney-derived extracellular vesicles (EVs). These nanoparticles can help clarify the status of the kidney and are a promising means of conducting a evaluating the renal function before transplantation (51).

As an effective means of improving the outcome of transplantation, nanotherapy before transplantation has attracted increasing attention. Zhu et al. used micelles to encapsulate rapamycin and modified the particles to enable targeting of endothelial and epithelial cells. The addition of targeted rapamycin micelles (TRAMs) to standard organ preservation solutions as a pre-transplant preservation regimen helps prevent downstream organ dysfunction and protects cells *in vitro* while preventing the vascular injury and fibrosis resulting from chronic rejection *in vivo* (52). PACE nanoparticles loaded with siRNA targeting CIITA were able to be delivered into organs *via* machine perfusion. Those nanoparticles enabled the efficient encapsulation and controlled release of siRNA, thereby facilitating the prolonged ongoing silencing of proteins of interest to protect organs from acute rejection after transplantation (53).

The importance of graft treatment before transplantation is self-evident, especially for DCD organs. We should thus seek out new renal-related nanoparticles that are more useful for evaluating the renal quality during perfusion, improving the microcirculation according to the evaluation results and then conducting NMP combined with NPs for targeted pre-transplantation treatment in order to further improve the graft prognosis.

## Nanotechnology in Artificial Biological Islets

### Islet Transplantation of Patients With Type 1 Diabetes Mellitus (T1DM)

T1DM is a heterogeneous autoimmune disease characterized by a decrease in the number of pancreatic β cells, resulting in insulin secretion dysfunction (54). For such patients, long-term glycemic control, disease management and complication prevention are important. Long-term use of exogenous insulin is an effective way to control blood glucose in such patients; however, while several options are currently available, adverse effects, such as injection site pain, inflammation and hypoglycemia, cannot be avoided (55). In contrast, islet transplantation is believed to be promising for refining physiological insulin secretion and glycemic control, thereby avoiding long-term dependence on exogenous insulin and associated complications; furthermore, it is less demanding than pancreas transplantation, as patients do not need to undergo major surgery (56, 57).

The Edmonton protocol confirmed islet transplantation as a promising approach for restoring endogenous insulin secretion in T1DM to control blood glucose; however, this regimen was unable to maintain insulin independence in the long term and still required the administration of strong immunosuppressive therapy (58). The side effects of long-term immunotherapy cannot be ignored, so how to further reduce the graft’s immune response within the recipient is the primary issue to address in order to improve the outcomes of islet transplantation.

### Microencapsulation

Encapsulation of islets with semipermeable membranes for immunosuppressive sequestration is a promising approach to achieving immunosuppressant-free transplantation (59). Lim and Sun were the first to use sodium alginate to encapsulate pancreatic islets, and their method prevented the direct contact of immune components with the islet cells without the use of immunosuppressive agents (ISAs), thereby reducing the immune response while not affecting the nutrient uptake by the pancreatic islets (60). Most subsequent studies achieved control over the cyst wall thickness and microcapsule size, improved the overall durability and reduced toxicity of the material itself for the islets of Langerhans by optimizing the encapsulated material (61–63). However, due to biocompatibility issues, while direct immune reactions with cells are avoided, the body’s nonspecific foreign body reaction to the microcapsules can lead to the proliferation of fibers, which can lead to disorders in the communication of the islets with external nutrients and eventually cause necrosis of the islets within the microcapsules (64, 65).

### Reducing the Immunoinflammatory Response Around Microcapsules

Anti-inflammatory-related drugs have benefits in reducing fibroproliferation, but the extent to which different drugs are effective is unknown, and adverse drug reactions caused by the way in which they are administered systemically are difficult to avoid. To this end, Dang et al. performed an intravital subcutaneous screen of 16 anti-inflammatory drugs and found that dexamethasone and curcumin had the strongest inhibitory effect on early inflammatory proteases and reactive oxygen species (ROS). Therefore, co-encapsulating the drug with the islets in alginate microcapsules, confirming that localized drug release inhibited fibrosis around the microcapsules can improve the control of blood glucose by the microencapsulated islets (64). This issue was similarly explored by Azadi et al., who designed an alginate/dextran-spermine microcapsule encapsulating pancreatic islets along with pentoxifylline (PTX), an anti-inflammatory drug. This therapeutic structure was not only able to effectively shield the islet cells inside the microcapsules but also alleviated the inflammatory response around the microcapsules and reduced fibroproliferation (66).

### Nanoparticles in Microencapsulation of Pancreatic Islets

Approaches to reduce the immunoinflammatory response around microcapsules, such as by optimizing the material to improve biocompatibility or by adding anti-inflammatory-related drugs, are reassuring for improving the outcomes of microcystic islet transplantation. Nevertheless, a large number of empty microcapsules may be left behind during islet microencapsulation, which increases the overall graft volume and leads to a stronger host immunoinflammatory response.

Espona et al. combined superparamagnetic iron oxide nanoparticles (SPIONs) with microfluidic technology to purify magnetically labeled pseudoislet-containing microcapsules in order to achieve automated screening of empty microcapsules, thereby reducing the probability of technical errors, and the microcysts that remained after screening greatly reduced the graft size (by over 75%) (67). Large volumes of capsules containing islets still need to be invested in order to achieve therapeutic purposes. The retrieval of these capsules after graft failure or loss of the graft function is an issue that needs to be urgently addressed. Delcassian developed a new encapsulated cell therapy with capsules that could be retrieved magnetically after transplantation by loading carboxylated iron oxide nanoparticles (NP-COOH) into encapsulated islet alginate hydrogels. This strategy preserved the encapsulated functionality (i.e. no need for immunosuppressive therapy for six to eight weeks) while guaranteeing the functionality of the transplanted islets, and no long-term leaching of the nanoparticles was noted (68).

### Layer-by-Layer (LBL) Polymer Self-Assembly

Although microencapsulation has proven effective in islet encapsulation, the issue of an excessive volume of microcapsules remains difficult to overcome due to the fabrication process (69, 70). An excessive microcyst volume can affect islet cell metabolic processes and limits the choice of transplant site, such as preventing transplantation to the most desirable site—the portal vein—and forcing the selection of sites such as the peritoneal cavity, where blood supply is not abundant (71), ultimately risking graft failure due to hypoxia. A much more refined encapsulation strategy is therefore needed to solve this problem.

**Figure 2:**
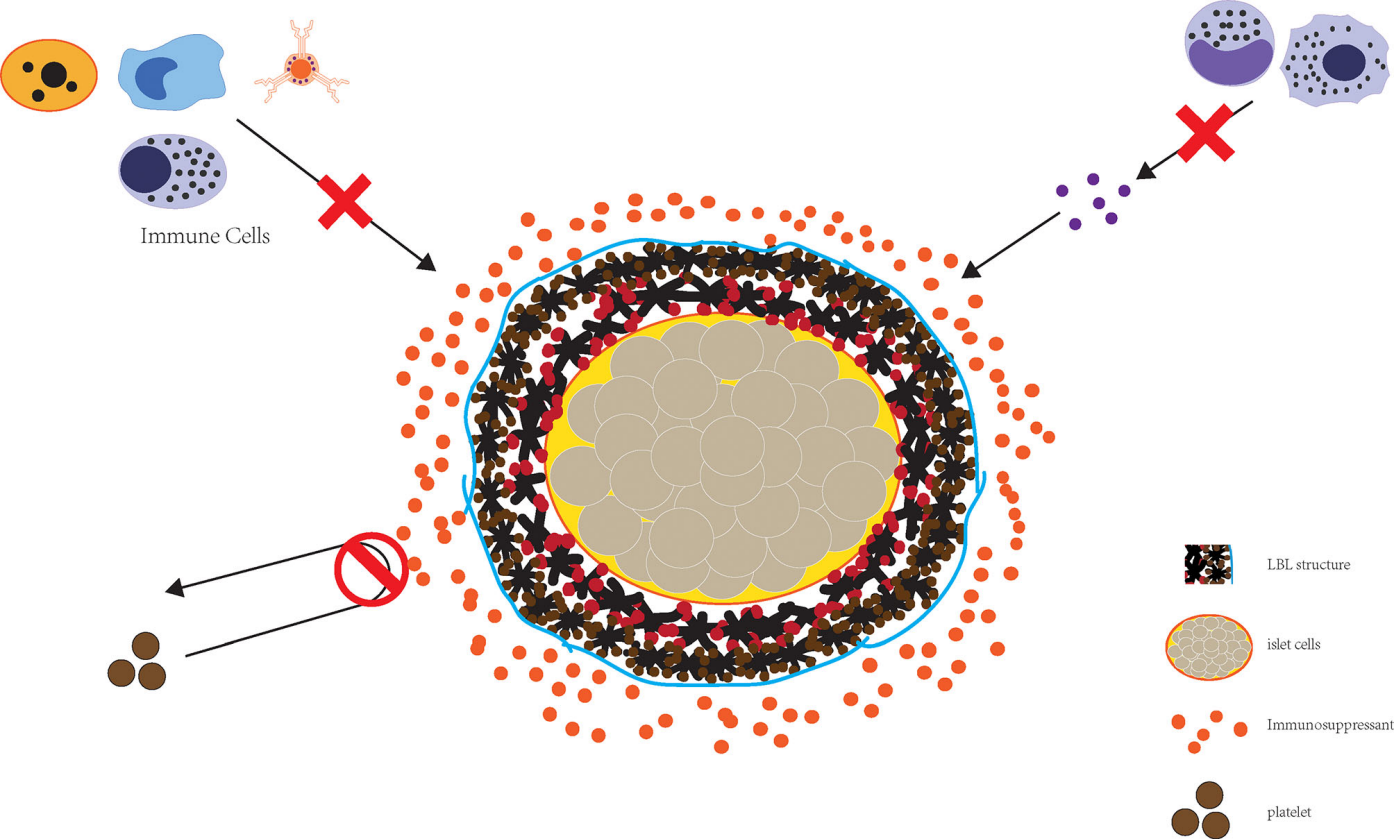
The combined application of LBL structure and immune preparation can effectively realize the destruction of islet cells by immunity.

LBL polymer self-assembly enables the preparation of nano-sized thin film coating and is a reasonable approach for downsizing encapsulated islets, and can effectively block the attack of immune components (**Figure 2**). Wilson et al. constructed a nano coating rich in PEG through LBL self-assembly of poly(L-lysine)-g-poly (ethylene glycol) (biotin) (PPB) and streptavidin (SA) and found that this coating did not cause a loss of islets while guaranteeing the immune isolation of islet cells. At the same time, nutrient exchange was not affected, nor was the islet viability or function (72). This encapsulation method can be performed in the nanometer range without concerns regarding diffusion barriers while at the same time greatly reducing the volume of transplanted islets, thereby allowing transplantation to the portal vein.

The feasibility of LBL islets was first validated in an NHP model by Haque et al. They covalently grafted three layers of PEG onto the surface of pancreatic islets *via* LBL technology, achieving a nano-shielding effect. The shielded islets were subsequently transplanted, and studies showed that the LBL islets were less immunogenic, did not have their activity or function affected, had a higher survival rate than non-LBL islets and suffered relatively little immune damage after the application of a glucocorticoid-free immunosuppressive drug regimen, demonstrating the feasibility of this transplantation strategy (73).

To improve the transplanted islet function, Kizilel constructed a nano thin multilayer film of biotin-PEG-NHS/SA/biotin-PEG-GLP-1 *via* the LBL method. This biofunctional multilayer film immobilized GLP-1 on the surface of pancreatic islets and not only improved the islet survival but also promoted insulin secretion, and the response time to glucose was not affected. This coating may reduce the number of islets required, which may help mitigate issues associated with the shortage of islet donors, while also decreasing the volume of the graft and thereby reducing the risk of an elevated portal pressure after transplantation (74).

### Reducing Inflammatory Damage

The instant blood-mediated inflammatory reaction (IBMIR) is a major cause of early and massive loss of intraportal transplanted islets and must be addressed in order to successfully initiate intraportal islet transplantation. This problem attracts the attention of a wide range of researchers, and many protocols are proved to be effective in reducing inflammatory injury (**Table 1**). Im et al. used 8-arm-PEG-catechol (PEG8) and N-hydroxysuccinimidyl-linked unfractionated heparin (UFH-NHS) to construct a dual-islet shielding system that effectively inhibited the transplanted islet mediated immune response and IBMIR, an effect that was further strengthened by adding the immunosuppressive agent FK506 (75). Haque et al. used polyethylene glycol plus heparin (heparin nano-shielded islets; HNSIs) in an LBL fashion to shield NHP islets, a protocol that not only blocks islet damage by immune cells but also functions in combination with most current immunosuppressive regimens, demonstrating its reliability in NHP models (76).

**Table 1:** The strategies of reducing inflammatory damage.

Author	Years	Method	Type of NPs	Model
**Im, Bok-Hyeon et al.**	2013	LBL	PEG8+UFH-NHS	Human
**Haque et al.**	2018	LBL	HNSI	HNP
**Haque et al.**	2018	LBL	Hb-PEG/Hep	HNP
**Izadi Zhila et al.**	2018	LBL	JAG1-PEG	Mice
**Kozlovskaya et al.**	2012	LBL	Tannic acid/N-vinylpyrrolidone	Human/NHP
**Abuid et al.**	2019	LBL	CONPs/alginate hydrogels	NHP
**Syed, Farooq et al.**	2018	MLBL	Chitosan/PSS	Human
**Gattás-Asfura et al.**	2020	CLBL	PAMAM	Mice

Islets remain vulnerable for most of the early period after transplantation due to the loss of their microenvironment. Restoring this microenvironment would thus be conducive to the islet survival and functional recovery. Therefore, Haque et al. constructed a hyperbranched polyethylene glycol (hb-PEG)/Heparin(Hep) nano-encapsule to promote the islet cell survival by shielding them from immune attack, a strategy that reduced IBMIR injury by mitigating complement activation and proinflammatory cytokine generation. This regimen in combination with immunosuppressive drugs had an even stronger effect of preserving the graft survival. The effect of hb-PEG/Hep nano-encapsules on the reestablishment of the islet microenvironment is not a direct effect, instead indirectly helping the islets recreate their own microenvironment by supporting the islet survival (77). Unlike the native environment under physiological conditions, transplanted islets are often in an inflammatory microenvironment, which directly affects the islet transplant survival. Izadi et al. developed a method for modifying the islet surface by coupling NHS groups on PEG to primary amines in the Jagged-1 (JAG-1) structure to immobilize JAG-1 on the surface of islets PEGylated, and these JAG1-PEG islets showed a greater ability to activate the Notch signaling pathway than untreated islets. Increasing CD4+, CD25+ and FOXP3+ cells as well as the IL-10 and TGF-β production while decreasing the INF-γ and TNF-α generation, which reverses the inflammatory microenvironment to an anti-inflammatory one, promotes the formation of an immunotolerant microenvironment that protects pancreatic islets from inflammatory injury (78).

### Exploring More Efficient LBL Technologies

Kozlovskaya et al. used a non-ionic hydrogen-bonded LBL technique to produce a novel and versatile cytoprotective coating that is more efficient than the conventional ionic LBL-based method. This method can be performed in a physiological state, and the resulting islets are uniformly coated with a non-toxic and non-immunogenic layer. They can stably exist *in vitro* without affecting the islet function or viability, supporting the modification of functional molecules and also playing a role in inhibiting proinflammatory cytokine synthesis (79).

An insufficient antioxidant capacity of islet cells and oxidative stress damage after transplantation are important causes of a poor function of transplanted islets as well as their death. Abuid et al. generated a nanoscale antioxidant coating through LBL assembly of cerium oxide nanoparticles (CONPs) and alginate hydrogels, a strategy that effectively and durably protected the encapsulated cells from ROS-mediated damage while preserving the cellular metabolism and ability to respond to glucose stimuli. The effect of protection correlates with the number of layers, and it has been shown that 12 layers of CONP/alginate hydrogels coatings have a stronger effect than 2 or 6 layers (80).

Syed et al. used electrostatic bonding technology to deposit two differently charged polymers (positively charged chitosan and negatively charged polystyrene sulfonate sodium salts [PSS]) for the multi-layer-by-layer (MLBL) encapsulation of islets. The MLBL islet activity and function were unaffected, and cells were protected from inflammatory cytokine damage. The transplanted islets showed an improved glucose tolerance and had a significant ability to reduce and stabilize blood glucose levels. A longer time after transplantation can also ensure the survival and morphology of islets and reduce the infiltration of immune cells (81).

The LBL method for constructing pancreatic islet cell coatings often utilizes electrostatic interaction, a promising and helpful approach, but the long-term stability and biocompatibility are issues that remain to be resolved. Gattás-Asfura et al. explored the engineering covalently-stabilized LBL (CLBL) coating approach, which used triethoxysilane with a functionalized poly (amino amides) (PAMAM) dendrimer to enhance the coating stability. Encapulation *via* the CLBL approach allowed for islet engraftment at sites such as the kidney capsule, with no significant difference from untreated islets in the ability to restore normoglycemia or stabilize blood glucose levels and also support islet re revascularization (82).

### Nanotechnology in Islet Xenotransplantation

Despite continuous advances in technology for islet encapsulation, the paucity of islet donors remains a barrier to the widespread use of artificial islets. Porcine insulin is structurally very similar to human insulin, and the porcine islet supply is not an issue at present, as access is very easy to achieve, making porcine islets a reliable replacement for human ones (83, 84). Porcine islets are derived from high-quality donors, unlike human donors, who are often elderly or deceased, and porcine islets can be modified by genetic engineering to reduce the immune response, resulting in an improved transplant success (85).

However, immune rejection remains a major obstacle to porcine islet xenotransplantation. Matsumoto et al. used alginate to wrap neonatal porcine islets and implanted them intraperitoneally in patients with T1DM in an experimental group. Their findings suggested undetected porcine endogenous retrovirus (PERV) infection in all subjects, with the HbA1c level maintained at <7%, thus significantly reducing hypoglycemic events. Longer-term maintenance was feasible, as no ISAs were applied, suggesting that immune shielding of porcine islets is an effective approach to address xenogeneic rejection (86). Lew et al. constructed exenatidel-loaded poly (lactic-co-glycolic acid) microspheres after determining the suitable drug-loading capacity and coencapsulated them in alginate microcapsules with porcine islets. The addition of exenatide reduced the rate of islet cell death and improved the stimulatory response to glucose, further enhancing the transplantation success of microencapsulated porcine islets (87).

LBL technology-based nanofilms as well as islet surface modification are an additional option for porcine islet xenotransplantation. Haque et al. combined SH-6-arm-peg-lipid and gelatin-catechol to construct a bilayer structured artificial extracellular matrix for encapsulating and stabilizing porcine islets against cell detachment. In parallel, the use of PEG-derivative encapsulation for islet camouflage reduced the overall immunogenicity and adhesion of serum albumin, fibronectin and immunoglobulin G (IgG). A cocktail immunosuppressant treatment regimen has been adopted after xenoimplantation, which can further inhibit the activation of immunity (83).

Neonatal porcine islet-like cell clusters (NPCCs) are popular because of their low price, ease of isolation and proliferative capacity, but they cause early loss of the graft and subsequent graft failure due to a transplant immune response, such as IBMIR or hyperacute rejection (88, 89). Nanoencapsulation can cope well with these problems, but it is still inescapable for damage in NPCCs encapsulation. Lee et al. focused on how to optimize nanoencapsulation for NPCCs to guarantee the quality of encapsulated islets and avoid unnecessary islet loss. They obtained more stable nanocapsules by inducing cross−linking between bifunctional psomes (NHS-/NH2 psomes) and also found that entrapment in F-10 medium at pH 7.3 could reduce the NPCC damage, while a higher yield of NPCCs could be achieved after adding 0.25% bovine serum albumin (90).

### Other Applications of Nanotechnology in Islet Transplantation

Nanotechnology attempts in optimizing islet transplantation are multidirectional and multiangular. Bilirubin is a powerful antioxidant, and its intravenous administration has been shown to reduce apoptosis and levels of inflammatory mediators in order to increase the graft survival in a murine transplant model. However, bilirubin is not readily soluble in water under physiological conditions and has a low bioavailability, and its repeated intravenous injections to maintain effective concentrations carry a risk of toxicity (91–93). Fullagar et al. encapsulated bilirubin into Pluronic F127-chitosan NPs to construct bilirubin nanoparticles. This nanodrug delivery strategy increased the bilirubin uptake and bioavailability by pancreatic islet cells and provided protection to pancreatic islet cells during times of oxidative stress (94). Huang et al. tried to control the insulin secretion process from the implanted islets. They entrapped islets using SA-PEI-melanin composite threadlike hydrogels, an approach that has good biocompatibility and enables effective immune shielding. The introduction of melanin nanoparticles can stimulate insulin secretion and further regulate insulin secretion *via* near-infrared (NIR), achieving a better control of blood glucose levels. Furthermore, they also have retrievable functions (95). Cheng et al. developed an efficient method of islet cryopreservation by combining microfluidic encapsulation systems, cold-responsive nanocapsules (CR-NCS) as well as calcium alginate hydrogel (CAH) embedding. Calcium alginate substitutes traditional toxic CPAs as islet cell cryoprotectants. CAH is responsible for protecting cells during the freeze-thaw process while providing a barrier to the transplanted islets. The islet cell function was preserved under this strategy, and a good glucose-lowering effect was still seen after implantation into diabetic rats. This method is expected to aid in the establishment of islet tissue banks, solve the issue of time lag between donors and recipients and help further mitigate the donor shortage (16).

Furthermore, pancreatic tissue engineering is a promising area for islet transplantation. Elham hoveizi et al. obtained nanofibrous PLA/Cs scaffold by electrospinning technique and incorporated zinc oxide nanoparticles (nZnO) into this 3D culture structure. The results demonstrate that this strategy can provide a favorable nano environment for Endometrial stem cells(EnCSs) differentiation into insulin-producing cells (IPCs) (96). Yung Chih Kuo’s group then found that pancreatic islet cells could be generated from IPCs in activin A-grafted gelatin-PLGA NP scaffold by controlling appropriate concentrations of activin A, LY294002, and retinoic acid (RA) (97).

For patients with chronic pancreatitis, on the one hand, long-term chronic pain seriously affects the quality of life of patients. On the other hand, chronic inflammation leads to progressive destruction of pancreatic islets and finally causes diabetes. Total-pancreatectomy (TP) with intraportal-islet-auto-transplantation (IAT) is one of the options for the treatment of chronic pancreatitis. This treatment modality provides pain relief and preserves meaningful islet function (98–100). The introduction of nanotechnology to improve the effect of autologous islet transplantation is a worthy direction for further optimization of this therapeutic approach.

From the use of alginate as a representative microencapsulation tool to the application of LBL technology for successful transplantation from allogeneic to xenografts, from NHP models to humans, from the optimization of encapsulation materials to emphasizing the encapsulation process, optimization of islets themselves, and islet preservation techniques, the continuous advancement and optimization of artificial biological islets have made islet transplantation an important strategy for the treatment of T1DM.

## Application of Nanoparticle Contrast Agent Imaging for Organ Transplantation

The rejection between donor and recipient of transplanted organs and the functional quality defects of transplanted islets may eventually lead to the failure of the whole transplant treatment program. Therefore, it is necessary to perform timely intervention according to the real-time state of the transplanted islets.

Real-time monitoring of the activity and function of the transplanted islets is critical for the treatment of T1DM. At present, the evaluation of the islet graft function mainly depends on the measurement of clinical biochemical indexes, including C-peptide levels, fasting and stimulated glucose levels and oral/intravenous glucose tolerance testing (101). However, these indexes can only indirectly reflect the functional status of islet grafts. Therefore, the establishment of a non-invasive real-time quantitative technique for monitoring the islet graft survival is important for the clinical application of islet transplantation (**Table 2**).

**Table 2:** Nanoparticles for organ transplant imaging.

Imaging mode	Types	Materials	Characteristics	Application model	Advantage	Disadvantage
**MRI**	SPIO	/	/	Patients with islet transplantation	/	Iron overload (spontaneous or induced)
**MRI**	SPIO	ATDM/CMDM/CMEADM/TMADM-01-05/DEAEDM	The nanoparticles are charged due to the substitution of the cationic terminal group of dextran	SD rats/diabetic nude mice	Effective introduction of cells	The iron content of liver is higher, and more artifacts occur
**MRI**	HSPIO	/	Anticoagulant activity	SD rats	IBMIR can be inhibited *in vivo* and *in vitro*, which can be quickly eliminated and will not accumulate in the liver	/
**FeMRA**	USPIO	Ferumoxytol	/	Patients with advanced renal transplantation	Safe and non-toxic, long blood half-life, good biocompatibility, can be swallowed by macrophages without cell activation	High concentrations of reagents may cause artifacts and cannot be directly applied to islets
**MRI**	USPIO	ferumoxtran-10	/	Patients with type 1 diabetes	Identify the highest risk individuals from occult pancreatitis to dominant diabetes	/
**MRI**	USPIO	Coated with amphiphilic polymer ([OE-PEG-COOH])	Bcl-2 functionalization of monoclonal antibodies	ICR mice	It can effectively label rodent islet cells and has good distribution and biocompatibility	High contrast medium concentration or inflammatory fluid infusion may cause low signal
**MRI**	USPIO	Ferumoxytol	A four-layer nanoshield with poly(ethylene) glycol (PEG, 2 layers), ferumoxytol, and heparin was formed on the pancreatic islets	a syngeneic mouse intraportal PIT model and a non-human primate intraportal PIT model	After multilayer modification of islet surface, Ferumoxytol USPIO can be directly used for the labeling of islets	/
**MRI**	dextran-coated iron oxide MNs	MN-siCaspase-3/MN-siB2M/MN-miR-216a Probe	Therapeutic/SiRNA nanoparticle probe for dual use	NOD-SCID mice	siRNA gene protection therapy and living body MRI feasibility of non-invasive monitoring of two-in-one combination of transplanted human islets in mice	The trend of particle aggregation hinders their large-scale synthesis
**MPI**	SPIO	dextran-coated Ferucarbotran SPIOs	/	NOD-SCID mice	An imaging method without depth attenuation and background tissue signal	Lack of background support in physiology and anatomy
**19F MRI, CT and US imaging**	perfluorocarbon nanoparticles	rhodamine-PFOB/rhodamine-PFPE	Multimode cell contrast agent	mice and rabbits	Combine the advantages of different imaging methods to overcome the limitations of a single imaging method	/
**19F MRI,** **Fluorescence, and Bioluminescence Imaging**	PLGA	poly(lactic-co- glycolic acid) (PLGA-NPs) with encapsulated perfluoro-15-crown-5-ether and the near-infrared fluorescent dye indocyanine green	Multimode cell contrast agent	rats	Any false positives in 19FMRI data can be eliminated	Most of them are complex and expensive, and lack a large number of clinical data verification

### Magnetic Resonance Imaging (MRI)

MRI is a reliable non-invasive imaging method for monitoring the efficiency of islet transplantation. It has outstanding clinical benefits, such as no harm to the patient due to ionizing radiation, a high representativeness of reuse, a high imaging resolution, deep tissue penetration and strong tomographic imaging ability. It has been widely used in the imaging-based monitoring of islet transplantation (102).

#### Superparamagnetic Iron Oxide (SPIO)

Because of the similar density of pancreas and liver on MRI, it is difficult to observe islet cells transplanted through the hepatic vein, so contrast agent is needed to distinguish them. Although islet cell labeling requires an effective uptake of MRI contrast agents, common commercial magnetic nanoparticles cannot be effectively introduced into cells, which has a great impact on imaging.

SPIO has become a widely used MRI contrast agent in clinical settings because of its low toxicity and high sensitivity (103). Koblas et al. showed that the labeling of islet cells with SPIO had no effect on the survival rate, activity or function of transplanted islets. Subsequently, Toso et al. confirmed for the first time in clinical practice that real-time imaging monitoring of islet cells by MRI with SPIO labeling was completely safe and feasible (104). Koichi et al. developed six kinds of magnetic iron oxide nanoparticles coated with different dextrans. The results suggested that these new positively charged nanoparticles might be useful MRI contrast agents for monitoring the islet quality after transplantation, thus confirming that MRI using SPIO nanoparticles contrast agent is a viable tool for *in vivo* monitoring of the islet quality (105).

Some studies on MRI have shown that, after labeling islet cells with these new positively charged nanoparticles, the T1- and T2-weighted imaging signals of islet transplantation sites in mice decreased significantly. However, this result is caused by the transplantation of islets into the subcapsular space of the kidney. If the islets are placed in the porta hepatis commonly used in clinical settings, more artifacts will occur due to the high iron content in the liver (106). This is an urgent problem to be solved in the application of this new positively charged nanoparticle technology in MRI.

In the study of Toso et al., the intensity of the images of three patients before islet transplantation was normal, and the iron-loaded islets were recognizable as low-intensity spots in the liver after transplantation. Another patient had a diffuse low-signal image on baseline liver MRI, so transplant-related changes could not be observed. Iron overload (spontaneous or induced) is the main obstacle to MRI with iron-containing contrast medium (104). Researchers have thus conducted a large number of studies on the modification, improvement and replacement of SPIO contrast media, with some breakthroughs made, and a large number of new SPIO contrast agents have been developed and put into clinical use.

IBMIR is one of the main inflammatory reactions that occurs after pancreatic islet transplantation through the hepatic portal vein, leading to the activation of platelets as well as the coagulation and complement systems. This platelet-amplified reaction creates activated platelets bind to the islets and contribute to a continuous fibrin formation, which can damage the transplanted islets, potentially causing an early function loss, and poses a serious challenge for clinical islet cell replacement therapy. To prevent such islet transplantation failure, a new type of heparin-immobilized HSPIO nanoparticles with anticoagulant activity was developed, and its chemical coupling can be used to camouflage the surface of islet cells. Yong et al. found that HSPIO nanoparticles were able to prevent IBMIR *in vivo* and *in vitro*, and the implantation of such labeled islets significantly reduced the blood glucose level of diabetic animals (107). Such HSPIO was also shown to be quickly eliminated after portal vein injection, did not accumulate in the liver, and was able to be used to track fixed islets, showing both MRI sensitivity and anticoagulant activity. It therefore seems to be a promising clinical choice for a new type of SPIO contrast agent with remarkable future prospects.

#### Ultra-Miniature SPIO (USPIO)

The biggest difference between USPIO and SPIO lies in their diameters: the diameter of USPIO is less than 50 nm, while that of SPIO is more than 50 nm. As a biodegradable preparation, USPIO is safer than SPIO and non-toxic, showing a long blood half-life, good biocompatibility, and an ability to be swallowed by macrophages without cell activation.

However, most reports thus far on USPIO have focused on the evaluation of kidney transplantation. Stoumpos et al. found that ferumoxytol-enhanced magnetic resonance angiography (FeMRA) was feasible and practical for vascular imaging in patients with advanced renal transplantation. It has the advantage of obtaining arteriography and venography findings at the same time without inducing nephrotoxicity (108). Maryam et al. found that ferumoxytol was not retained in renal allografts in patients with acute rejection, and that renal allografts with acute rejection showed a prolonged T^2^ * value compared with non-rejection allografts (109).

It was recently reported for the first time that the USPIO nanoparticle Ferumoxtran-10 can be used in the clinical treatment of islet inflammation in patients with T1DM. In addition, it is a feasible contrast agent for the commercial use of USPIO, receiving approval from the US Food and Drug Administration (FDA) in 2005 (110). Bin et al. found that using a new type of Bcl-2-functionalized PEG-USPIO as a molecular imaging agent had great potential for monitoring islets or other cells *in vivo* (111). Sang-Man et al. found that the labeling effect of USPIO-based MRI was not directly suitable for clinical islet transplantation, so they evaluated the feasibility of ferumoxytol in islet MRI by multi-layer surface modification. They found that multi-layer islet surface modification was a promising choice for portal vein concave islet MRI (112). Therefore, after proper surface modification, the ordinary commercially available USPIO should be able to be used to evaluate the status of islet transplant patients as a promising new contrast agent.

#### Therapeutic Diagnostic Imaging

The term “therapeutic diagnostics” was first proposed by Funkhoer et al. in 2002 (113). Treatment diagnostic imaging is combined with MRI diagnostic imaging and related intervention therapy. It is used to identify specific targets by imaging, design relevant reagents for the targets and visualize them and monitor the treatment response, so as to minimize any effects on normal tissue (114). It can analyze specific information by combining the genome and proteome, enabling the design of a personalized treatment for organ transplantation, which has great prospects and significance for clinical applications in the future.

Wang et al. designed an MR probe by coupling therapeutic siRNA with glucan-coated SPIO and incubated the probe with it before islet transplantation. The results showed that islets transplanted under the renal capsule had a better survival by reducing the expression of caspase-3 in the MN-siCaspase-3 group, while hyperglycemia caused by T cell attack in the MN-siB2M group was significantly delayed (102, 103). Ping et al. synthesized a nano-therapeutic agent composed of magnetic nanoparticles (MNs) coupled with siRNA molecules targeting genes harmful to islet grafts. The combined therapeutic effect provided by an RNA interference technique with an *in vivo* MRI diagnosis is expected to improve the results of islet transplantation significantly in patients with T1DM (115).

Recently, a new kind of nanoparticle was synthesized: MNs targeting phosphatase and tensin homologue (PTEN) coupled to miR-216a. These nanoparticles can down-regulate the expression of PTEN to promote the proliferation of islet β-cells in T1DM animal models (116). However, while these new nanoparticles have the advantage of penetrating the biofilm barrier, particle aggregation trends hinder their large-scale synthesis (117).

### Magnetic Particle Imaging (MPI)

MPI is a newly emerging real-time monitoring and imaging method that can directly perform imaging using magnetized iron oxide nanoparticles. It has strong specificity, high sensitivity and safe translation (118). The sensitivity of MPI is 108 times higher than that of the proton magnetization seen in MRI (119). At the same time, MPI is safe and translatable because its carrier iron oxide nanoparticles are biocompatible (120) and do not produce ionizing radiation, and magnetic fields can be used with no threat to patient safety (121).

Ping et al. described the first application of MPI in the monitoring of transplanted islets. SPIO was used to label islets separated from baboons (*Papio hamadryas*). The results showed that MPI was an imaging method without depth attenuation or a background tissue signal, which is suitable for imaging transplanted islet grafts. However, MPI lacks the background support of physiology and anatomy depictions, which is the main issue limiting its clinical application (122).

### Multimodal Imaging

The simple use of an imaging mode has obvious limitations, and the observed research results cannot be completely scientifically representative. For example, performing MRI just once results in a low sensitivity, its contrast medium is somewhat toxic, islets must be prelabeled before transplantation, and it is difficult to distinguish between living and dead islets. In contrast, Bioluminescence Imaging (BLI) and Fluorescence Imaging (FI)have disadvantages of poor tissue penetration, a short elimination period, low spatial resolution, propensity to be affected by the internal environment, and a tendency to show weak signals (102). These issues lead to a bottleneck in the clinical promotion of single imaging techniques and fail to objectively reflect the real situation. Therefore, it is very necessary to develop a multi-mode imaging platform that combines the advantages of different imaging methods and overcomes the limitations of individual methods.

Barnett et al. labeled human islet organs with PFOB and PFPE nanoparticles and visualized the islets under the renal capsule of mice and rabbits by MRI, ultrasound and computed tomography (123). The combination of specific and quantitative Fmur19 MRI and sensitive and convenient optical imaging can provide supplementary information to support the distribution and survival of islet grafts. Based on poly (lactic acid-glycolic acid) (PLGA-NPs) wrapped in perfluoro-15-crown-5-ether and NIR fluorescent dye indocyanine green, Gálisová successfully developed a three-mode imaging platform for the *in vivo* examination of islet transplantation (124).However, most of these multi-mode nanoparticles are more complex and expensive than conventional single modality nanoparticles, and the effects of these nanoparticles on transplanted islet cells and human body and their stability need to be explored.

## Drug Delivery of Nanoparticles for Organ Transplantation

To improve the success rate of organ cell transplantation, patients need to take ISAs to alleviate the immune response. However, systemic administration of ISAs is associated with serious side effects; for example, the oral and intravenous administration of tacrolimus may lead to systemic toxicity, which greatly reduces the long-term success rate of transplantation (125). The emerging method of nanodelivery may improve the disadvantages of systemic drug delivery *via* the targeted delivery of ISAs to specific organs and tissues.

### Improving the Pharmacokinetics and Delivery Efficiency of ISAs

For drugs with a narrow therapeutic index or low bioavailability, intervention with nanoparticle technology can potentially optimize the pharmacokinetics and delivery efficiency. In the early stage of developing transport platforms for ISAS, Edgar et al. found that cyclosporine was needed after renal transplantation, and the pharmacokinetics of cyclosporine were affected by a high lipid value, suggesting the relatively minor influence of a fat-rich meal on the absorption of cyclosporine from Sandimmune Neoral is advantageous (126).. Indeed, Ritschel et al. found that the bioavailability of cyclosporine was significantly improved when it was used as a microemulsion particle in clinical trials of kidney and liver transplant recipients (127).

At the same time, some studies have found that the release of the mTOR inhibitor rapamycin from poly(ethylene glycol)-b-poly(ϵ-caprolactone) (PEG-PCL) micelles *in vitro* has remarkable benefits (128). A large number of studies have shown that the pharmacokinetic characteristics and delivery efficiency of ISAs can be adjusted by changing the size, shape, surface charge and composition of nanoparticles (129–131). The size of nanoparticles is of great significance in guiding the delivery of drugs to specific transplanted organs (132). After intravenous injection, nanoparticles of different diameters are quickly removed by the kidney, liver and spleen (133–135) or preferentially gathered in the liver and spleen (136, 137). Studies have shown that nanoparticles are injected into the arterial blood supply of organs before transplantation, so the selection of an appropriate particle size will directly affect the intraorgan penetrance (138). Nanoparticles with an appropriate diameter can better meet their target, thereby improving the delivery efficiency of ISAs.

The shape of nanoparticles is also a key factor for determining the half-life of nanoparticles in the circulation. Gentile et al. found that discoidal particles are more likely to marginalize and adhere to the endothelium than spherical particles because of their specific tumbling and edge dynamics (139). Geng et al. found that the circulation time of phyllo-micelles nanoparticles in the body is longer than that of spherical nanoparticles. At the same time, some studies have shown that nanoparticles can affect the internalization of cells due to the change in the phagocytosis rate depending on their external shape, thus improving the pharmacokinetic characteristics and delivery efficiency of ISAs (134).

However, the most important parameter is the surface composition of nanoparticles. Poly(lactic-co-glycolic acid) (PLGA) is a family of biodegradable copolymers, the ultimate degradation products are lactic acid and glycolic acid and easily metabolized by the body. Therefore, it is frequently used clinically as nanoparticles carrying ISAs. ISAs encapsulated within them have the advantages of stability, long circulation time and guided drug release (140). Studies have shown that encapsulating ISAs, such as cyclosporine, tacrolimus and rapamycin, in liposome nanoparticles, such as polyethylene glycol-PLGA (PEG-PLGA), for a controlled release can stabilize the inherent variability of their pharmacokinetics (127, 141–143). High-density lipoproteins (HDLs) can also be used for the surface composition of small, dynamic nanoparticles carrying low doses of statins in order to alter the activity of the immune response through the internalization of macrophages (144). Nanogels coated with intracellular adhesion molecule-1 (ICAM-1) antibodies consisting of a mixture of dextran and lysozyme were found to provide effective drug delivery *in vitro* and *in vivo* (145).

Although we can improve the pharmacokinetics and delivery efficiency of ISAs by artificially setting the above parameters, it is extremely important to ensure the stability of these parameters during drug delivery. Results thus far have shown that the chemical composition of nanoparticles can be maintained using inert metal, graphene oxide or composite surfactant (146–148). As nanoparticles tend to target out-of-organ aggregation after collision, which greatly reduces their delivery efficiency, we need to reduce the aggregation effect in order to improve the stability of nanoparticles (149). These limitations associated with the clinical application are difficult problems that must be resolved in the future.

### Targeted Drug Delivery Mode and Mechanism of Nanoparticles

**Figure 3:**
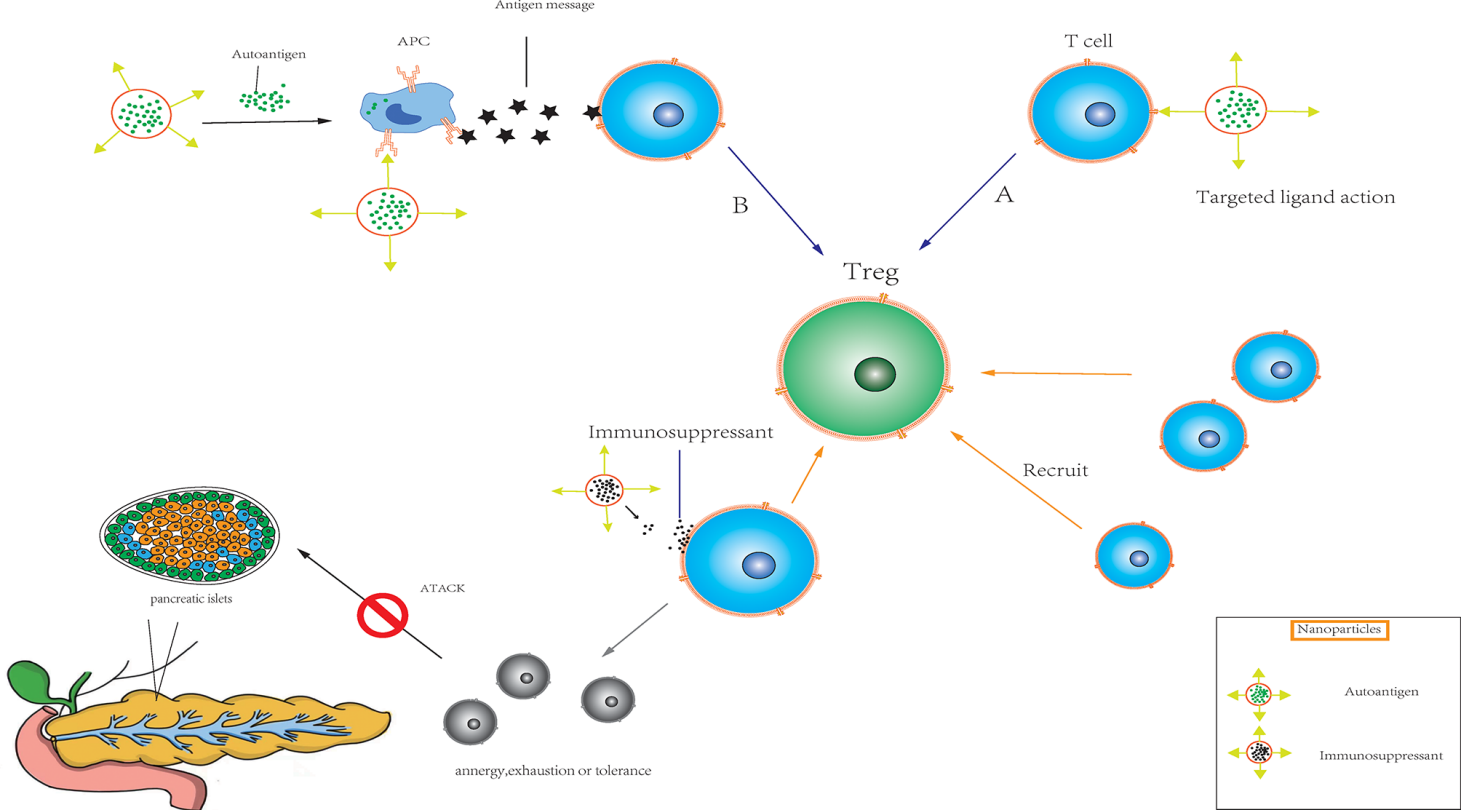
Immunomodulatory nanoparticles **(A)** Nanoparticles target non-phagocytic cells such as T cells in transplanted organs (pancreas, etc.) by exploring ligand-T cell receptor (TCR) interactions. **(B)** Nanoparticles target antigen-presenting cell (APC) through the continuous release of their own antigens or the surface coupling of ligands, and induce antigen presentation to APC. The combination of the two modalities can quickly and effectively activate the target organ to produce regulatory T cells in the absence of inflammatory signals, and recruit a large number of T cells. At the same time, under the combined action of ISAs released by nanoparticles, resulting in T cell anergy, exhaustion or tolerance.

Nanoparticles are often used as movement-targeting agents because they can deliver drugs directly to different locations in tissues and/or cells. Since endogenous autoantigen presentation may have a synergistic effect with targeted inhibition, nanoparticles that deliver ISAs to the target site are a feasible therapeutic strategy. Nanoparticles can target phagocytes (such as dendritic cells) while carrying ISAs or genetic material to achieve therapeutic effects. Alternatively, drug therapy can be achieved by exploring the interaction of ligand-T cell receptor (TCR), which targeting non-phagocytic cells (such as T cells). Nanoparticles can also be used to facilitate the continuous release of autoantigens or to target antigen-presenting cells (APCs) through surface coupling of ligands. In the absence of inflammatory signals, nanoparticles can induce the presentation of self-antigens to APCs, resulting in T cell anergy, failure or tolerance (**Figure 3**). In addition, nanoparticles can transfer antigens and ISAs through surface delivery and/or soluble delivery, resulting in antigen-specific immune regulation (150, 151). Shirali et al. upregulated PD-L1 on dendritic cells *via* the targeted delivery of mycophenolic acid by nanoparticles to prolong the survival time of mouse allografts (152). Shah et al. used an allogeneic skin transplantation model to optimize the transplantation tolerance of the PLG nanoparticle-peptide delivery platform, suggesting that the design of perfect targeted nanoparticles requires suitable immune cell populations (153). Studies have shown that carboxylated 500-nm biodegradable PLG nanoparticles (surface-coupled or encapsulated homologous diabetic peptides) can quickly and effectively restore the tolerance of non-obese diabetic (NOD) mice (154). Nanoparticles have been used to transmit tolerance-causing molecules and cellular antigens, thereby inducing tolerance phenotypes of dendritic cells and promoting the formation of Treg cells *in vivo* (155). Based on the injectable dual-sized microparticle (MP) platform, the immune system can be reeducated in an antigen-specific manner. These particles can be subcutaneously injected into NOD mice to prevent diabetes (156).

In addition, the binding of antigen peptides and red blood cells to antibody fragments *via* nanoparticles promoted antigen processing by scavenging apoptotic cells mediated by bionic red blood cells in a mouse T1DM model (157). Thomas et al. protected islet donors from non-human primates (NHPs; baboons) by silencing a gene responsible for inducing apoptosis (Caspase3), which was connected to a small interference RNA on MNs (158). Targeted antigen transmission can induce immune tolerance, and nanoparticles can be used to improve the effect of antigen therapy. However, antigen transmission alone may not be sufficient to overcome strong immune rejection and chronic inflammation after replacement with organ cells. The administration of tolerance-inducing drugs in the absence of antigens may lead to non-specific immunosuppression, while the administration of antigens alone may not be effective for treating multi-antigen heterogeneous diseases, such as T1DM. Nanoparticles can be designed to deliver antigens presented on MHC molecules to promote effective antigen presentation and thereby improve specificity and induce inhibition of the T cell phenotype. In the absence of any supportive costimulatory ligands, the treatment of NOD mice with these nanoparticles can inhibit the occurrence of diabetic symptoms *via* the interaction between particles and self-reactive T cells.

## Conclusion

Continuous advances in transplantation technology are improving the survival of patients with end-stage organ failure, and the addition of nanotechnology is undoubtedly exciting, given the quandary faced by transplantation technology. Whether it be in combination with pretransplant organ preservation *via* machine perfusion, or as artificial biological islets undergoing constant optimization, or through nanoimaging for the *in vivo* survival assessment of transplanted organs, or in the optimization of traditional immunosuppressive drug pharmacokinetics, nanotechnology has been intimately involved in addressing the problems faced by traditional transplantation, and these applications are constantly proving to be extremely promising.

We herein reported advances made in nanotechnology that we feel are inspirational, mainly by discussing the applications of nanotechnology in kidney transplantation as well as pancreatic islet transplantation. However, at present, the application of these findings in the clinical setting is not yet feasible, and the long-term efficacy and safety of nanotechnology need further observation and discussion. The development of nanomaterials and continuous advances in the strategies used for transplantation will ultimately lead to a more favorable prognosis for transplant patients.

## Author Contributions

All authors contributed to the writing and editing of the manuscript, contributed to the article, and approved the submitted version.

## Funding

This work was supported in part by Medical Science and Technology Project of Zhejiang Province (2021PY083), Program of Taizhou Science and Technology Grant (20ywb29), Major Research Program of Taizhou Enze Medical Center Grant (19EZZDA2), Open Project Program of Key Laboratory of Minimally Invasive Techniques and Rapid Rehabilitation of Digestive System Tumor of Zhejiang Province (21SZDSYS01, 21SZDSYS09) and Key Technology Research and Development Program of Zhejiang Province (2019C03040).

## Conflict of Interest

The authors declare that the research was conducted in the absence of any commercial or financial relationships that could be construed as a potential conflict of interest.

## Publisher’s Note

All claims expressed in this article are solely those of the authors and do not necessarily represent those of their affiliated organizations, or those of the publisher, the editors and the reviewers. Any product that may be evaluated in this article, or claim that may be made by its manufacturer, is not guaranteed or endorsed by the publisher.

## Abbreviations

IAK, implantable artificial kidney; DCD, donated after circulatory death; HMP, hypothermic machine perfusion; SCS, static cold storage; NMP, normothermic machine perfusion; APACs, antiplatelet and anticoagulants; IRI, ischemia-reperfusion injury; EVLP, *ex vivo* lung perfusion; ECs, endothelial cells; TLN, thrombalexin; cRGD, cyclic RGD; EVs, extracellular vesicles; TRAMs, targeted rapamycin micelles; ISAs, immunosuppressive agents; ROS, reactive oxygen species; PTX, pentoxifylline; SPIONs, superparamagnetic iron oxide nanoparticles; LBL, layer-by-layer; PEG8, 8-arm-PEG-catechol; UFH-NHS, N-hydroxysuccinimidyl-linked unfractionated heparin; HNSIs, heparin nano-shielded islets; JAG-1, Jagged-1; CONPs, cerium oxide nanoparticles; MLBL, multi-layer-by-layer; CLBL, covalently-stabilized LBL; PERV, porcine endogenous retrovirus; NPCCs, neonatal porcine islet-like cell clusters; CR-NCS, cold-responsive nanocapsules; CAH, calcium alginate hydrogel; mTOR, mammalian target of rapamycin; PEG-PCL, poly(ethylene glycol)-b-poly(ϵ-caprolactone); PLGA, Poly (lactic-co-glycolic acid); PEG-PLGA, poly(ethylene glycol)- Poly (lactic-co-glycolic acid); HDLs, high density lipoproteins; ICAM-1, intracellular adhesion molecule-1; TCR, T cell receptor; APC, antigen presenting cells; PD-L1, programmed cell death-Ligand 1; PLG, poly (D,L-lactide-co-glycolide); NOD, non-obese diabetic; MP, microparticle; T1DM, type 1 diabetes mellitus; NHP, non-human primates; MN, magnetic nanoparticles; MHC, major histocompatibility complex; MRI, magnetic resonance imaging; SPIO, superparamagnetic iron oxide; IBMIR, immediate blood-mediated inflammatory response; USPIO, ultra-miniature superparamagnetic iron oxide; FeMRA, ferumoxytol-enhanced magnetic resonance angiography; FDA, food and drug administration; PTEN, phosphatase and tensin homologue; MPI, magnetic particle imaging; BLI, bioluminescence Imaging; FI, fluorescence Imaging; PFOB, perfluorooctylbromide; PFPE, perfluoropolyethers.
